# Supplementary material for: Glycan-based biological degraders targeting the cytokine immune axis
Source: Commun Biol. 2026 Apr 11;9:530. doi: 10.1038/s42003-026-10001-9 (PMC13083842; doi:10.1038/s42003-026-10001-9)
Supplement: Supplementary file 1 — Supplementary Information [file 42003_2026_10001_MOESM1_ESM.pdf]

Supplementary Information

Supplementary Table 1: Thermodynamic parameters of BioDeg variants were assessed by nanoDSF. Listed are onset temperature and melting points, which are defined as the temperature at which 50% of the protein are unfold. Data shown is the mean of three technical replicates ± SD.

	**Onset [°C]**	**$T_{m,1}$ [°C]**	**$T_{m,2}$ [°C]**
**Siltuximab(-TGN)**			
Siltuximab	61.20 ± 0.35	67.05 ± 0.06	79.46 ± 3.20
Siltuximab-TGN 1:1	62.20 ± 0.26	67.03 ± 0.07	80.78 ± 0.24
Siltuximab-TGN 1:5	61.70 ± 0.16	66.60 ± 0.05	80.75 ± 0.11
Siltuximab-TGN 1:10	n.d.	65.45 ± 0.10	80.10 ± 0.48
Siltuximab-TGN 1:25	57.49 ± 0.47	63.42 ± 0.13	77.96 ± 0.13
**Tocilizumab(-TGN)**			
Tocilizumab	63.23 ± 0.08	68.60 ± 0.11	80.24 ± 0.23
Tocilizumab-TGN 1:1	62.27 ± 0.29	68.58 ± 0.06	80.31 ± 0.15
Tocilizumab-TGN 1:5	62.49 ± 0.61	68.36 ± 0.11	80.01 ± 0.11
Tocilizumab-TGN 1:10	61.61 ± 0.31	68.05 ± 0.06	79.53 ± 0.07
Tocilizumab-TGN 1:25	59.84 ± 0.49	67.00 ± 0.03	78.23 ± 0.16
**VHH_IL6(-TGN)**			
VHH_IL6	50.83 ± 0.58	58.87 ± 0.24	
VHH_IL6-TGN 1:1	50.51 ± 1.01	58.62 ± 0.21	
VHH_IL6-TGN 1:5	48.65 ± 0.48	58.03 ± 0.27	
VHH_IL6-TGN 1:10	45.27 ± 2.10	56.85 ± 0.39	
**sIL6R(-TGN)**			
sIL6R	44.05 ± 0.79	50.95 ± 0.19	
sIL6R-TGN 1:1	44.25 ± 0.90	51.11 ± 0.14	
sIL6R-TGN 1:5	44.25 ± 0.26	51.81 ± 0.20	
sIL6R-TGN 1:10	43.90 ± 0.81	51.97 ± 0.15	
sIL6R-TGN 1:25	42.67 ± 2.27	51.27 ± 0.15	

Supplementary Table 2: Kinetic parameters of BioDeg variants determined by SPR for binding to IL-6 and IL-6R. The table provides an overview of association (k_{on}) and dissociation (k_{off}) rate constants as well as binding affinities (K_D). Additionally, quality metrics for the 1:1 binding model are listed, including transport correction (tc) and Chi² (goodness of fit between model and sensorgram data. IL-6 and IL-6R were immobilized on a CM5 sensor chip via amine coupling. Analytes were injected using a multi-cycle kinetic format. Measurements were performed on a Biacore T200 using the corresponding control and evaluation software.

	k_{on} [1/Ms]	k_{off} [1/s]	K_D	tc	Chi^2 [RU^2]
**Siltuximab(-TGN)**					
Siltuximab	4.45×10^5	$< 10^{-5}$	< 22 pM	7.65×10^{14}	0.58
Siltuximab-TGN 1:1	5.22×10^5	$< 10^{-5}$	< 19 pM	1.52×10^{10}	1.75
Siltuximab-TGN 1:5	3.75×10^5	$< 10^{-5}$	< 27 pM	8.61×10^{10}	0.33
Siltuximab-TGN 1:10	2.78×10^5	$< 10^{-5}$	< 36 pM	7.76×10^9	0.05
Siltuximab-TGN 1:25	2.21×10^5	$< 10^{-5}$	< 45 pM	1.65×10^{10}	0.01
**Tocilizumab(-TGN)**					
Tocilizumab	2.65×10^5	1.80×10^{-5}	68 pM	3.31×10^{14}	0.05
Tocilizumab-TGN 1:1	2.63×10^5	$< 10^{-5}$	< 38 pM	5.33×10^{12}	0.13
Tocilizumab-TGN 1:5	2.52×10^5	2.36×10^{-5}	93 pM	2.32×10^{14}	0.07
Tocilizumab-TGN 1:10	2.35×10^5	4.98×10^{-5}	212 pM	2.48×10^{14}	0.04
Tocilizumab-TGN 1:25	1.91×10^5	6.26×10^{-5}	328 pM	1.39×10^{14}	0.05
**VHH_IL6(-TGN)**					
VHH_IL6	5.69×10^5	6.28×10^{-5}	110 pM	2.37×10^{14}	0.07
VHH_IL6-TGN 1:1	6.11×10^5	7.42×10^{-5}	121 pM	3.65×10^{14}	0.08
VHH_IL6-TGN 1:5	2.06×10^5	4.17×10^{-5}	203 pM	3.08×10^{15}	0.004
VHH_IL6-TGN 1:10	1.54×10^5	4.08×10^{-5}	265 pM	7.68×10^7	0.006
**sIL6R(-TGN)**					
sIL6R	2.68×10^5	0.035	131 nM	1.56×10^{12}	0.07
sIL6R-TGN 1:1	2.29×10^5	0.038	166 nM	4.77×10^{12}	0.06
sIL6R-TGN 1:5	1.65×10^5	0.038	230 nM	5.92×10^{11}	0.03
sIL6R-TGN 1:10	7.57×10^5	0.032	428 nM	7.35×10^{11}	0.06
sIL6R-TGN 1:25	no binding				

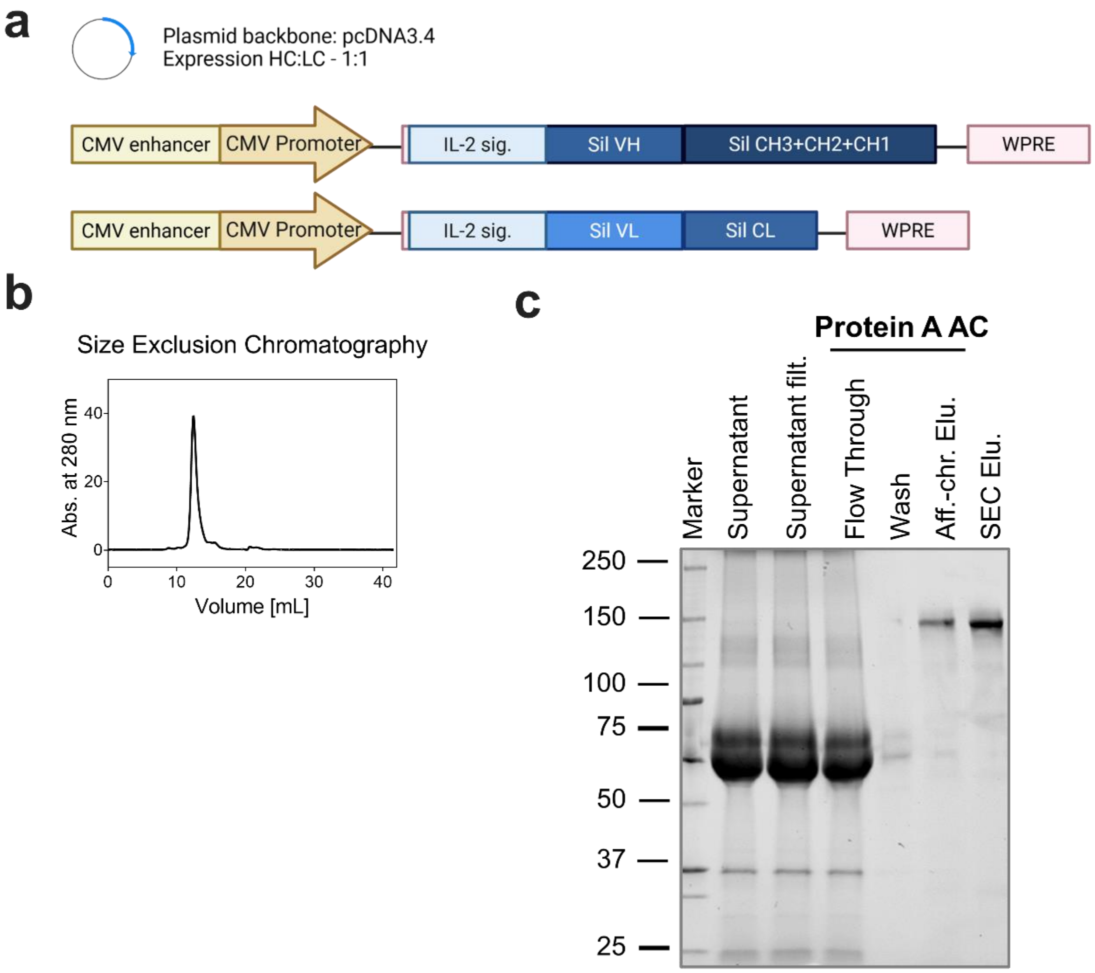

Supplementary Figure 1: Siltuximab Design & Production (a) Plasmid Design for Siltuximab heavy and light chain[1]. Inserts displayed here were cloned into a pcDNA3.4 vector for expression in HEK293T cells. **(b)** Purification via Protein A affinity chromatography and SEC with **(c)** SDS-PAGE analysis of each purification step.

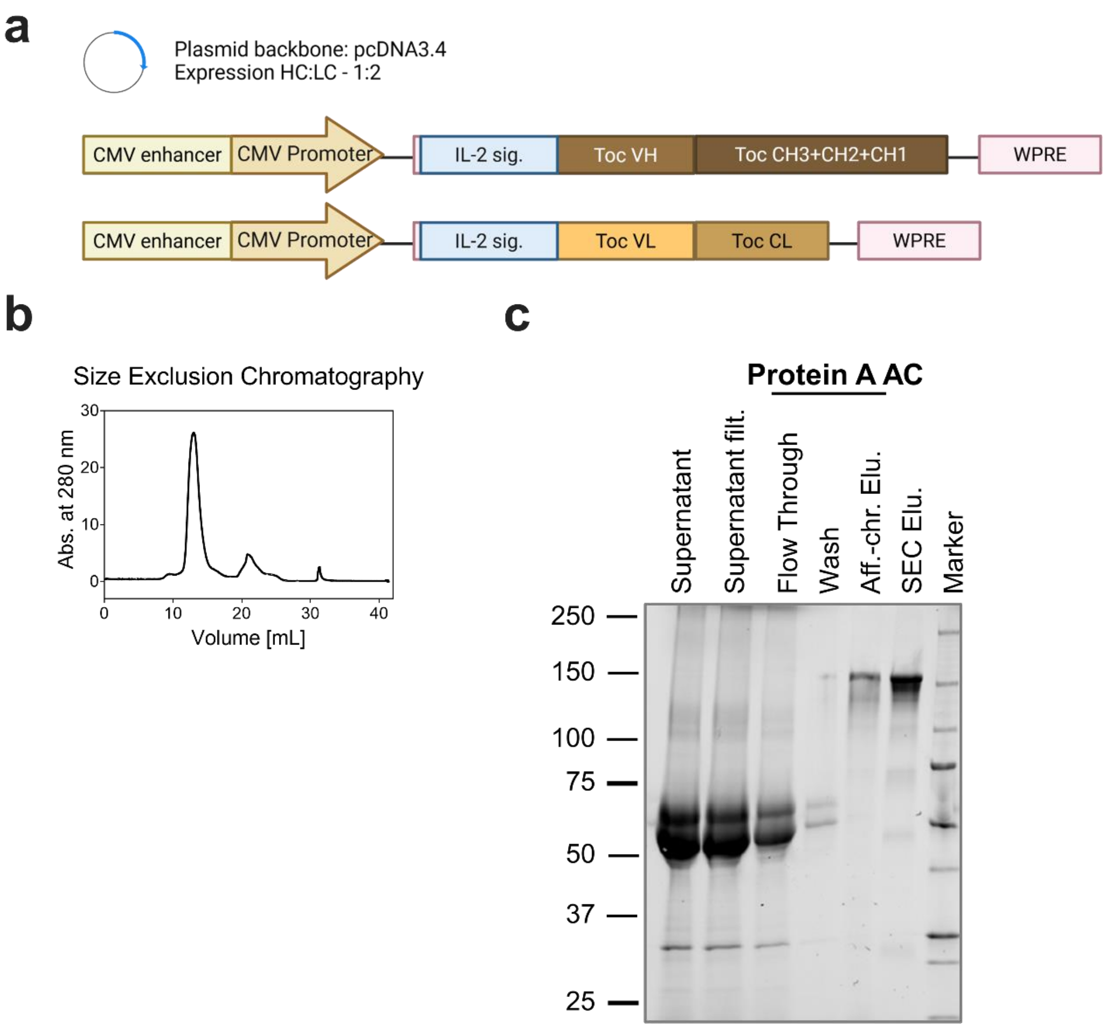

Supplementary Figure 2: Tocilizumab Design & Production (a) Plasmid Design for Tocilizumab heavy and light chain[1]. Inserts displayed here were cloned into a pcDNA3.4 vector for expression in HEK293T cells. **(b)** Purification via Protein A affinity chromatography and SEC with **(c)** SDS-PAGE analysis of each purification step.

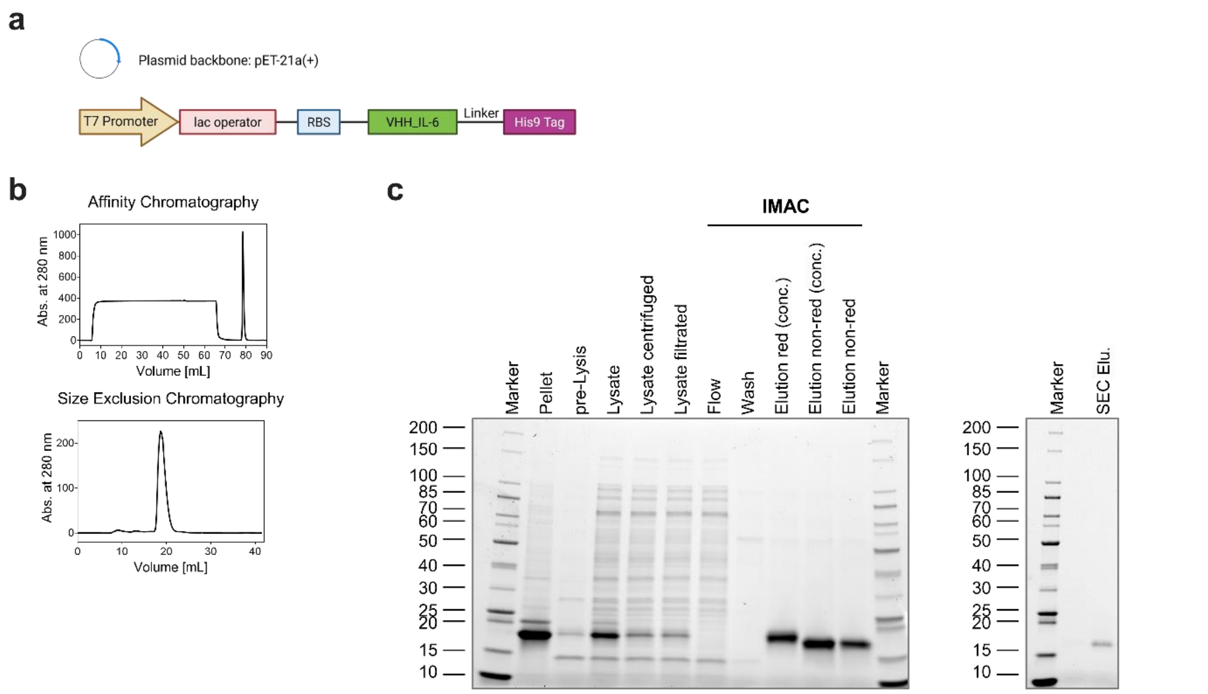

Supplementary Figure 3: VHH against IL-6 Design & Production (a) Plasmid Design for VHH_IL-6 with a His9 Tag[1]. Inserts displayed here were cloned into a pET21a(+) vector for expression in SHuffle® T7 (Express) *E. coli.* **(b)** Purification via Nickel Immobilized Metal Affinity Chromatography (Ni-IMAC) and SEC with **(c)** SDS-PAGE analysis of each purification step.

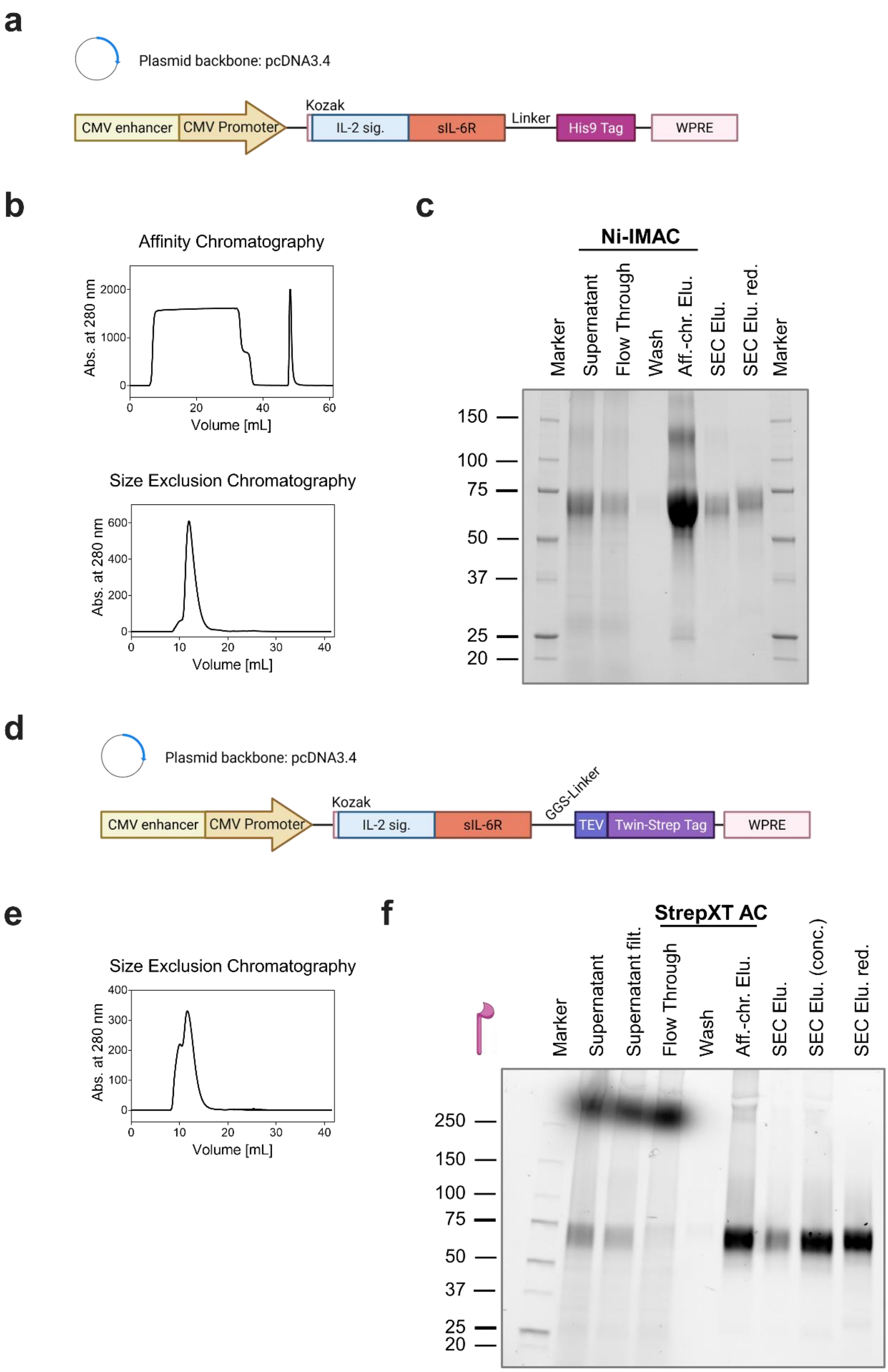

Supplementary Figure 4: sIL-6R Design & Production (a) Plasmid Design for sIL-6R with a His9-Tag[1]. Inserts displayed here were cloned into a pcDNA3.4 vector for expression in EXPI293F cells. **(b)** Purification via Ni-IMAC and SEC with **(c)** SDS-PAGE analysis of each purification step. **(d)** Plasmid Design for sIL-6R with a Twin-Strep-Tag and TEV cleavage site[1]. Inserts displayed here were cloned into a pcDNA3.4 vector for expression in EXPI293F cells. **(b)** Purification via StrepTactin XT based affinity chromatography and SEC with **(c)** SDS-PAGE analysis of each purification step.

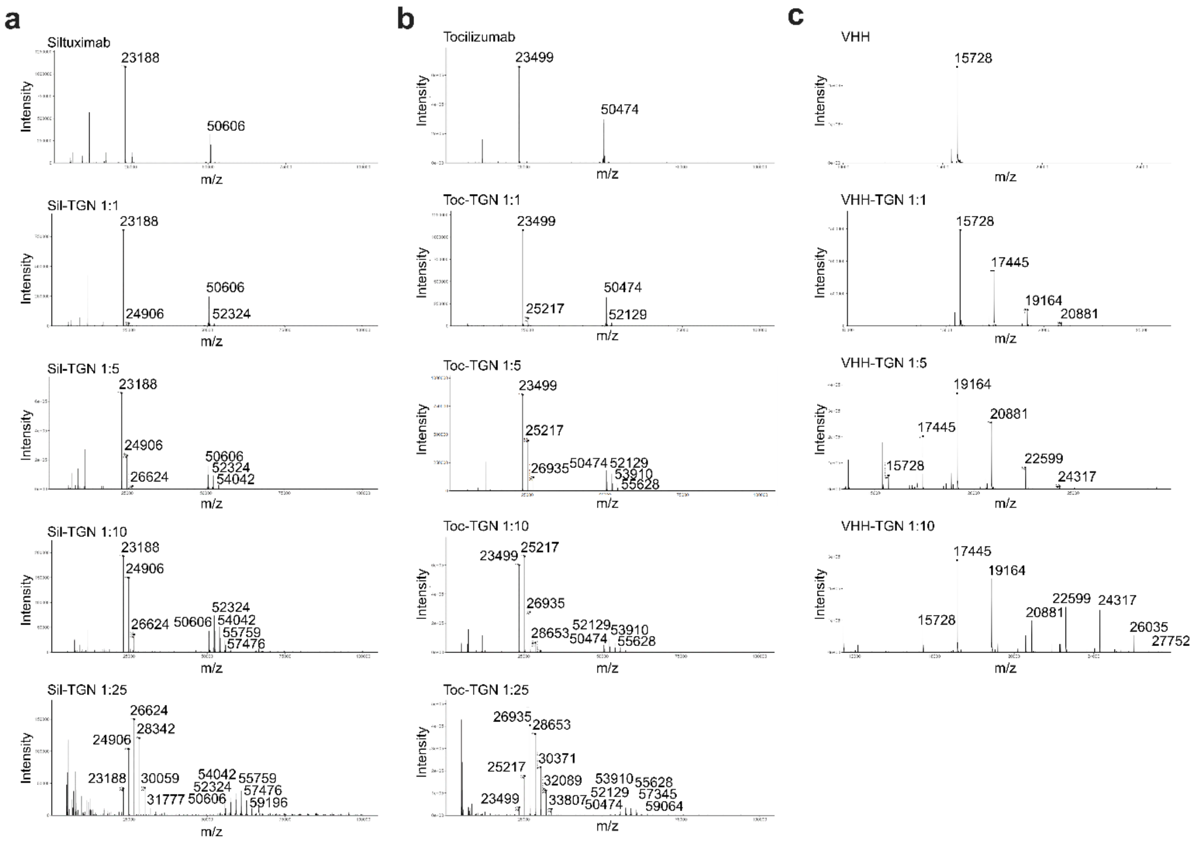

Supplementary Figure 5: Intact mass analysis of BioDeg constructs by ESI-QF mass spectrometry. (a) siltuximab(-TGN), **(b)** tocilizumab(-TGN) and **(c)** VHH_IL-6(-TGN). Shown are deconvoluted mass spectrum showing all detected intact mass species across the measured mass range (a, b) or zoomed view of the deconvoluted spectrum displaying a mass window relative to the most abundant intact mass peak, highlighting relevant species (c).

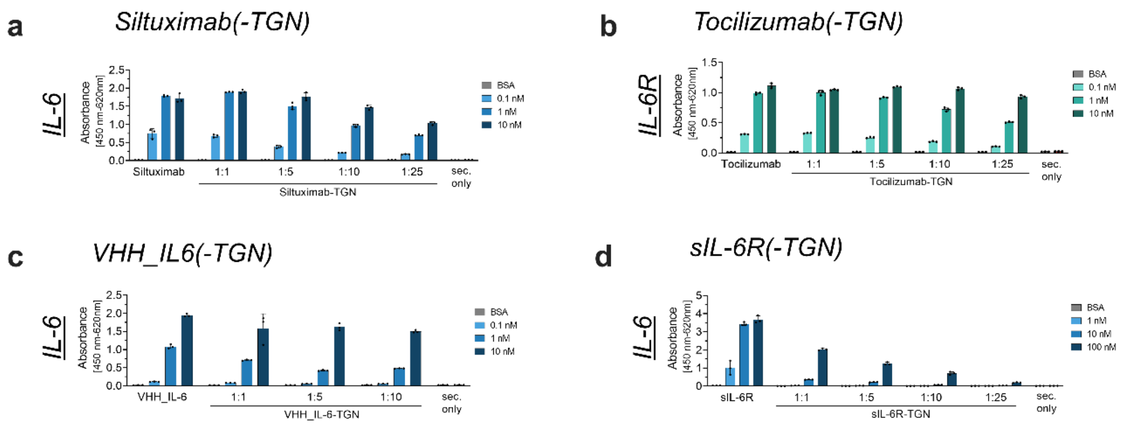

Supplementary Figure 6: Binding of BioDeg constructs to IL-6/ IL-6R assessed by indirect ELISA. Binding analysis of four BioDeg constructs: **(a)** Siltuximab-TGN, **(b)** Tocilizumab-TGN, **(c)** VHH_IL-6-TGN and **(d)** sIL-6R-TGN. Detection was performed using anti-His-HRP for VHH_IL-6 and sIL-6R constructs, and anti-human-HRP for antibody-based constructs (Siltuximab, Tocilizumab), with absorbance measured at 450 nm (reference: 620 nm) after TMB development. A sec. only control was included to assess background signal from anti-human/ anti-His-HRP binding in the absence of sample incubation. Assays were conducted in PBS. Data are shown as mean ± SD of technical triplicates.

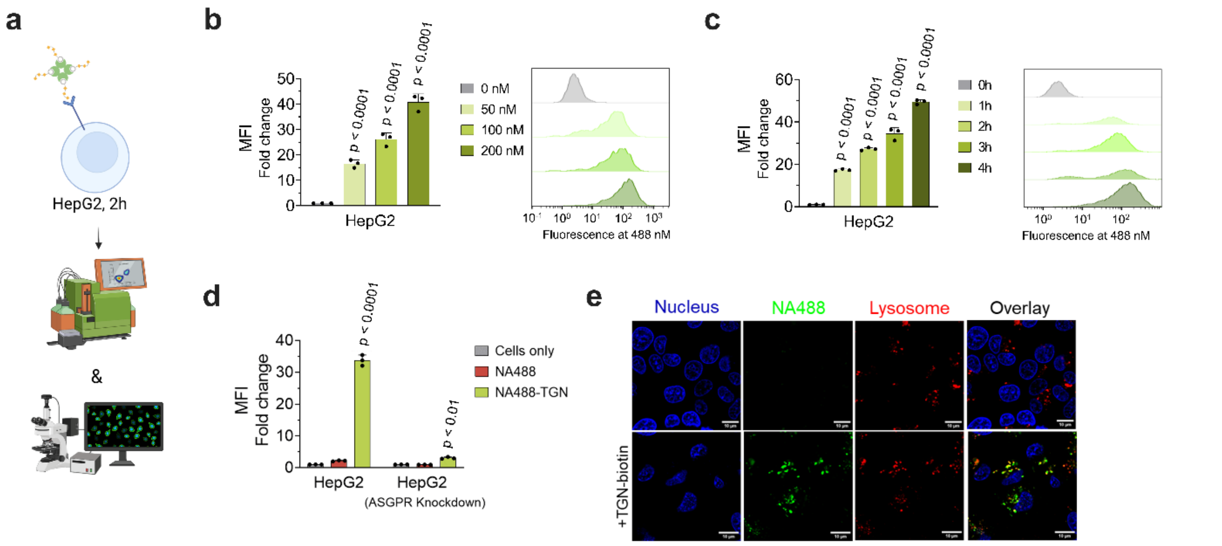

Supplementary Figure 7: Establishment of Tri-GalNAc (TGN) uptake in HepG2 cells. (a) Schematic representation of uptake assay using fluorescent Neutravidin (Oregon Green 488, NA488) in complex with TGN (NA488:TGN, 1:3)[2]. Flow cytometry quantification of TGN uptake in HepG2 cells in a **(b)** concentration-dependent and **(c)** time-dependent manner. **(d)** Uptake of TGN in ASGPR-Knockdown HepG2 cells, including NA488 alone as control (100 nM, 2 h incubation). Data are mean ± SD of three biological replicates. **(e)** Confocal microscopy showing internalized NA488 (green) +/- TGN, lysosomes (red; LysoTracker™), and nuclei (blue; NucBlue™). Assay was performed at 100 nM NA488 (300 nM TGN) with 2 h incubation time. Scale bars = 10 µm. **Statistical analysis:** One-Way ANOVA (b,c)/ Two-Way ANOVA (d) analysis with Dunnet's multiple comparisons test was used. < 0.1 (*), < 0.01(**), < 0.001 (***) and < 0.0001 (****) indicate significant differences between untreated and treated cells. *MFI* = mean fluorescence intensity. Fold change was calculated as normalization to untreated cells.

a

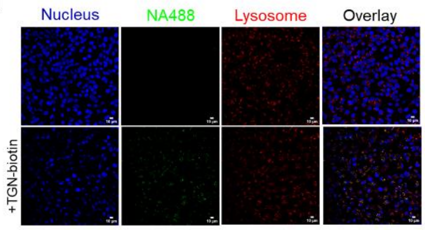

NA488	Pearson	M1	M2
+TGN	0.607	0.610	0.638

b

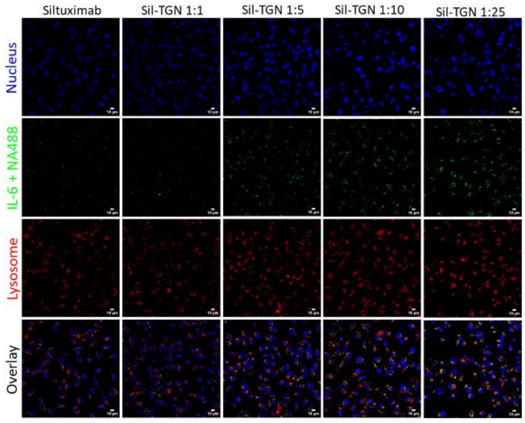

Siltuximab	Pearson	M1	M2
-TGN 1:1	0.478	0.693	0.77
-TGN 1:5	0.727	0.811	0.795
-TGN 1:10	0.690	0.835	0.941
-TGN 1:25	0.778	0.874	0.811

c

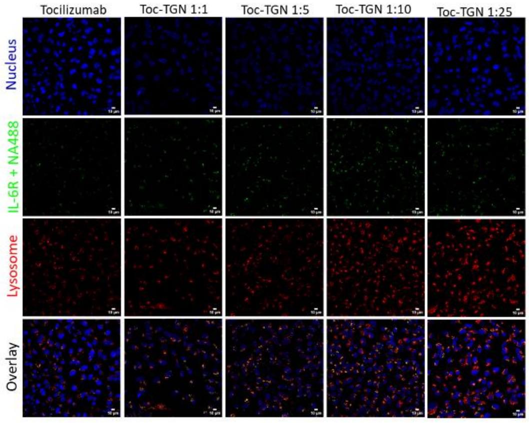

Tocilizumab	Pearson	M1	M2
-TGN 1:1	0.697	0.772	0.855
-TGN 1:5	0.706	0.807	0.855
-TGN 1:10	0.617	0.566	0.846
-TGN 1:25	0.657	0.832	0.833

d

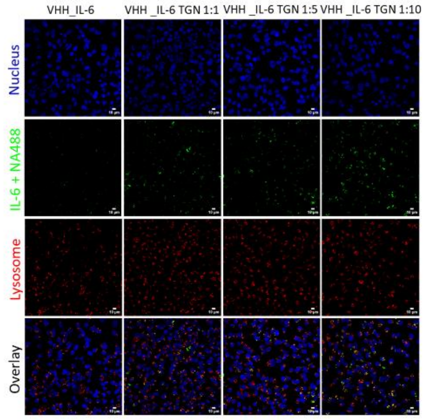

VHH_IL-6	Pearson	M1	M2
-TGN 1:1	0.345	0.811	0.501
-TGN 1:5	0.529	0.614	0.690
-TGN 1:10	0.534	0.912	0.570

e

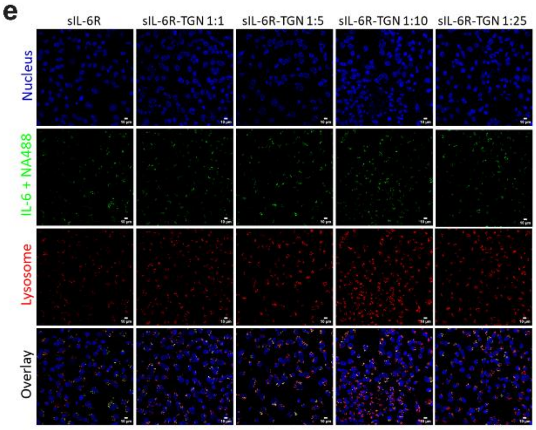

sIL-6R	Pearson	M1	M2
-TGN 1:1	0.724	0.784	0.816
-TGN 1:5	0.669	0.811	0.847
-TGN 1:10	0.630	0.670	0.896
-TGN 1:25	0.629	0.887	0.797

Supplementary Figure 8: Live-cell confocal microscopy showing intracellular localization of IL-6 (green), lysosomes (red; LysoTracker™), and nuclei (blue; NucBlue™). Images show > 10 cells each. Pearson correlation coefficient and Manders‘ coefficients were calculated using the JaCoP Plugin in ImageJ. Thresholds were set manually. M1 = ch01 (red) overlapping ch02 (green); M2 = ch02 (green) overlapping ch01 (red). Scale bars = 10 µm. **(a)** NA488 +/- TGN-biotin; **(b)** Siltuximab (-TGN) + IL-6; **(c)** Tocilizumab(-TGN) + IL-6R; **(d)** VHH_IL-6(-TGN) + IL-6; **(e)** sIL-6R(-TGN) + IL-6.

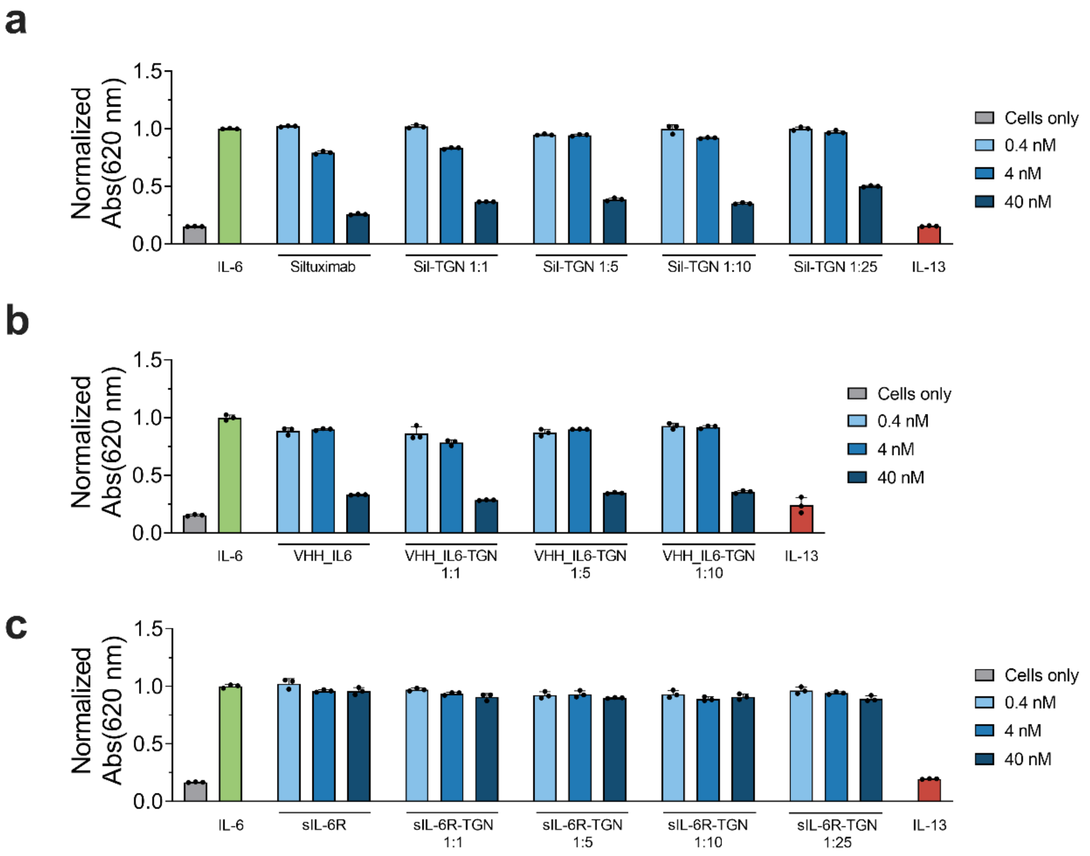

Supplementary Figure 9: IL-6 signaling in HEK-Blue™ IL-6 reporter cells. HEK-Blue™ IL-6 cells were stimulated with IL-6 (100 ng/mL) in complex with **(a)** Siltuximab(-TGN), **(b)** VHH_IL-6(-TGN), or **(c)** sIL-6R(-TGN) at the indicated concentrations (0.4 nM, 4 nM, 40 nM). IL-13 (100 ng/mL) was included as a negative control. SEAP activity was measured after 20 h of incubation using QUANTI-Blue™ reagent. Absorbance was recorded at 620 nm. Data represent mean ± SD of biological triplicates.

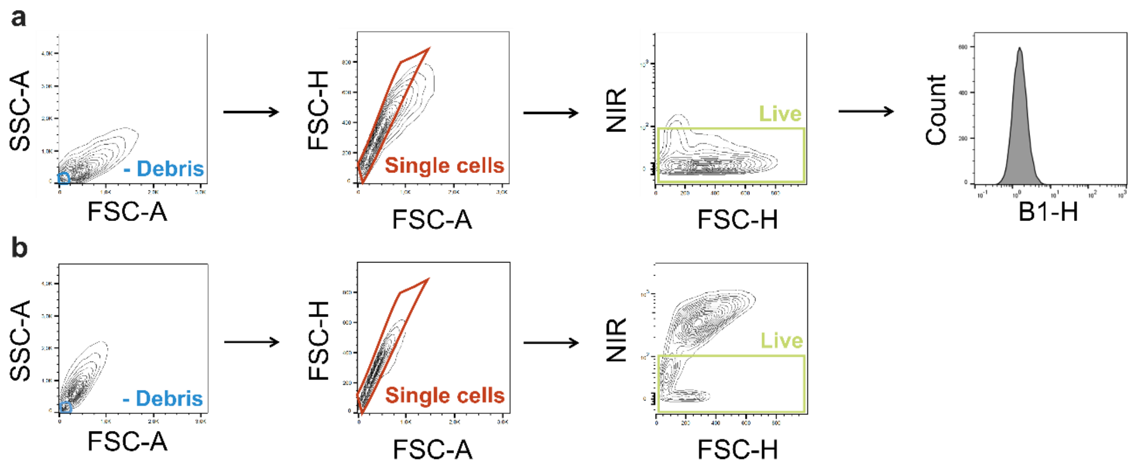

Supplementary Figure 10: Gating strategy for flow cytometry analysis in HepG2 cells. (a) Gating of viable HepG2 cells; **(b)** gating of dead cells based on near-infrared (NIR) viability dye exclusion. Initial gating was performed using forward scatter (FSC) and side scatter (SSC) to exclude debris and identify the main cell population. Singlet discrimination was applied via FSC-A vs. FSC-H gating to eliminate doublets and aggregates. Viability gating based on NIR staining allowed exclusion of dead cells. A histogram illustrates the fluorescence intensity (e.g., NA488-IL-6 signal) in the gated, viable single-cell population. Fluorescence thresholds were defined using untreated and unstained controls. All gating steps were applied consistently across biological replicates.

Fig. 3a

AF488 (ASGPR)

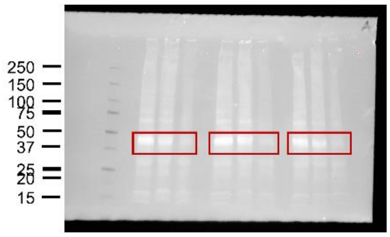

Fig. 3h

AF680 (IL-6) | AF546 (GAPDH) | AF680 (IL-6)

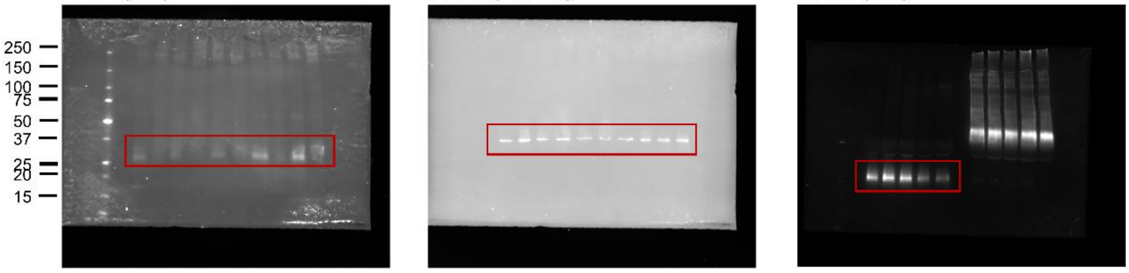

Fig. 5h

AF680 (sIL-6R) | AF546 (GAPDH) | AF680 (sIL-6R)

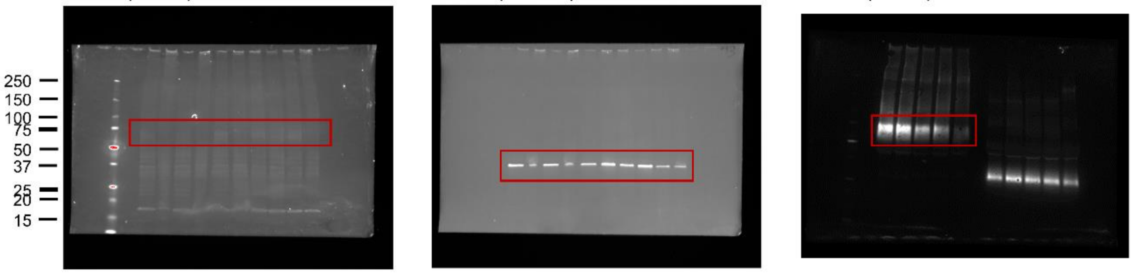

Fig. 7e

AF680 (IL-6) | AF546 (GAPDH) | AF680 (IL-6)

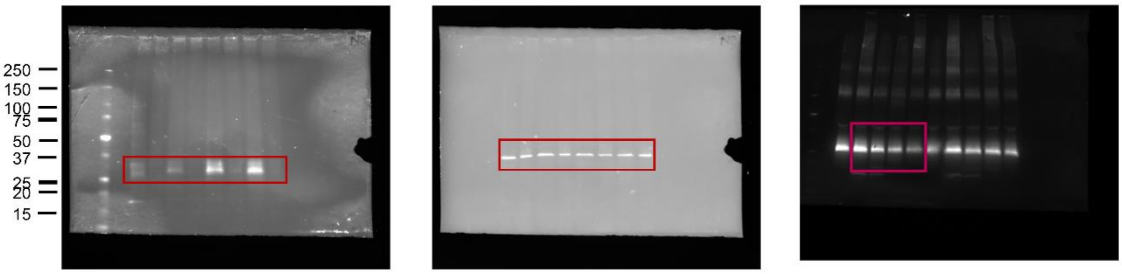

Fig. 10e

AF680 (IL-6) | AF546 (GAPDH) | AF680 (IL-6)

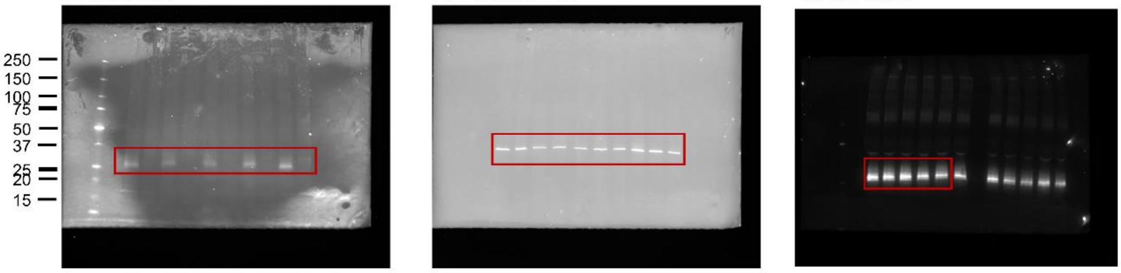

Supplementary Figure 11: Full, unprocessed Western blot membranes corresponding to the cropped images shown in Figures 3-10. The regions used in the main figures are indicated by red boxes.

Fig. 2b
Stain-free SDS PAGE gel

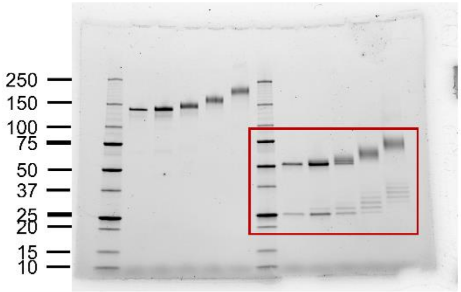

Fig. 2b
Stain-free SDS PAGE gel

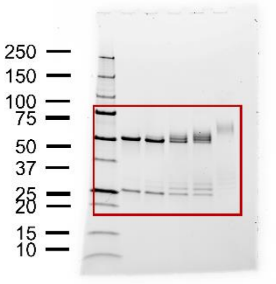

Fig. 6b
Stain-free SDS PAGE gel

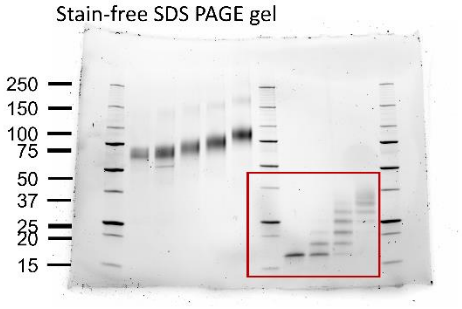

Fig. 8b
Stain-free SDS PAGE gel

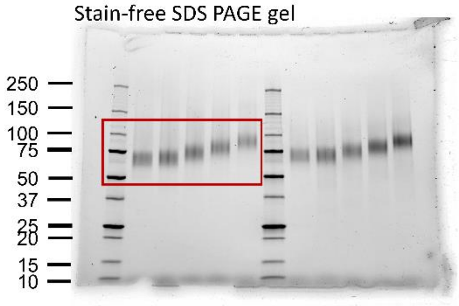

Supplementary Figure 12: Full, unprocessed SDS PAGE gels corresponding to the cropped images shown in Figures 2-8. The regions used in the main figures are indicated by red boxes.

Siltuximab(-TGN)

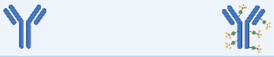

GPR		1:1	1:5	1:10	1:25
		0.20	1.76	3.91	14.87
**Binding (K_D)**					
IL-6:	< 22 pM	< 19 pM	< 27 pM	< 36 pM	< 45 pM
sASGPR:	-	+	++	+++	++++
**Uptake FC (normalized to unmodified)**					
IL-6	1	1.09	2.22	2.61	3.95
**Degradation WB (normalized to unmodified)**					
IL-6	1	1.05	0.87	0.57	0.47

Tocilizumab(-TGN)

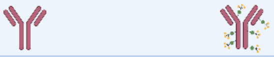

GPR		1:1	1:5	1:10	1:25
		0.35	2.51	4.75	15.36
**Binding (K_D)**					
IL-6:	68 pM	< 38 pM	93 pM	212 pM	328 pM
sASGPR:	-	+	++	+++	++++
**Uptake FC (normalized to unmodified)**					
IL-6R	1	1.14	1.60	2.08	2.32
**Degradation WB (normalized to unmodified)**					
IL-6R	1	0.99	0.70	0.63	0.38

VHH_IL-6(-TGN)

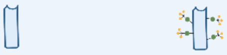

GPR		1:1	1:5	1:10
		0.57	2.16	4.25
**Binding (K_D)**				
IL-6:	110 pM	121 pM	203 pM	265 pM
sASGPR:	-	+	++	+++
**Uptake FC (normalized to unmodified)**				
IL-6	1	1.78	2.26	4.83
**Degradation WB (normalized to unmodified)**				
IL-6	1	0.90	0.55	0.41

sIL-6R(-TGN)

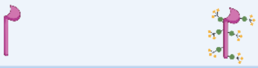

Binding (K_D)		1:1	1:5	1:10	1:25
IL-6:	131 nM	166 nM	230 nM	428 nM	-
sASGPR:	-	+	++	+++	++++
**Uptake FC (normalized to unmodified)**					
IL-6	1	1.14	1.37	1.37	1.34
sIL-6R	1	2.26	6.42	7.91	9.30
**Degradation WB (normalized to unmodified)**					
IL-6	1	1.05	0.91	0.95	0.89

Supplementary Figure 13: Overview of BioDeg Performance in interaction studies and *in vitro* assays. Comparison between unmodified binder molecules and the TGN-modified variants.

1. Created in BioRender. *Safarian, S. (2025)* https://BioRender.com/qlelnew.
2. Created in BioRender. *Safarian, S. (2025)* https://BioRender.com/bfvbs3q.
