# Supplementary material for: Glycan-based biological degraders targeting the cytokine immune axis
Source: Commun Biol. 2026 Apr 11;9:530. doi: 10.1038/s42003-026-10001-9 (PMC13083842; doi:10.1038/s42003-026-10001-9)
Supplement: Supplementary file 2 — Description of Additional Supplementary Materials [file 42003_2026_10001_MOESM2_ESM.pdf]

Description of Additional Supplementary Files

File name: Supplementary Data

Description: All numerical source data underlying the graphs and charts
