# Supplementary material for: Glycan-based biological degraders targeting the cytokine immune axis
Source: Commun Biol. 2026 Apr 11;9:530. doi: 10.1038/s42003-026-10001-9 (PMC13083842; doi:10.1038/s42003-026-10001-9)
Supplement: Supplementary file 4 — Reporting Summary [file 42003_2026_10001_MOESM4_ESM.pdf]

nature portfolio

Corresponding author(s): Schara Safarian

Last updated by author(s): Feb 11, 2026

Reporting Summary

Nature Portfolio wishes to improve the reproducibility of the work that we publish. This form provides structure for consistency and transparency in reporting. For further information on Nature Portfolio policies, see our Editorial Policies and the Editorial Policy Checklist.

Statistics

For all statistical analyses, confirm that the following items are present in the figure legend, table legend, main text, or Methods section.

n/a	Confirmed	
☐	☒	The exact sample size (*n*) for each experimental group/condition, given as a discrete number and unit of measurement
☐	☒	A statement on whether measurements were taken from distinct samples or whether the same sample was measured repeatedly
☐	☒	The statistical test(s) used AND whether they are one- or two-sided<br>*Only common tests should be described solely by name; describe more complex techniques in the Methods section.*
☒	☐	A description of all covariates tested
☒	☐	A description of any assumptions or corrections, such as tests of normality and adjustment for multiple comparisons
☐	☒	A full description of the statistical parameters including central tendency (e.g. means) or other basic estimates (e.g. regression coefficient) AND variation (e.g. standard deviation) or associated estimates of uncertainty (e.g. confidence intervals)
☐	☒	For null hypothesis testing, the test statistic (e.g. *F*, *t*, *r*) with confidence intervals, effect sizes, degrees of freedom and *P* value noted<br>*Give P values as exact values whenever suitable.*
☒	☐	For Bayesian analysis, information on the choice of priors and Markov chain Monte Carlo settings
☒	☐	For hierarchical and complex designs, identification of the appropriate level for tests and full reporting of outcomes
☐	☒	Estimates of effect sizes (e.g. Cohen's *d*, Pearson's *r*), indicating how they were calculated

Our web collection on statistics for biologists contains articles on many of the points above.

Software and code

Policy information about availability of computer code

Data collection	For data acquisition, fluorescence microscopy images were captured using Leica LAS X (v4.8.2.29567, Leica Microsystems). Western blot signals were recorded with Image Studio (v5.2, Bio-Rad). Plate reader measurements were performed on a MARS Plate Reader (v4.20, BMG LABTECH), while thermal stability measurements were carried out using PR.ThermControl (v2.3.1, NanoTemper Technologies). Surface plasmon resonance (SPR) data were acquired with Biacore T200 Control Software (v3.2.1, Cytiva).
Data analysis	For data analysis, flow cytometry data were processed using FlowJo™ (v10.1, Treestar), and statistical analyses and graphing were performed with GraphPad Prism® (v7, GraphPad Software). Structural modeling and AI-based predictions were conducted with AlphaFold™ 3 (v3, DeepMind), and molecular visualization was performed using ChimeraX (v1.9, UCSF Resource for Biocomputing, Visualization, and Informatics). Image processing and analysis were carried out with ImageJ (v1.54g, open-source, Schindelin et al., 2012), including subcellular colocalization analysis using the JACoP plugin (S. Bolte & F. P. Cordelières, 2006). Thermal stability data evaluation was performed using PR.StabilityAnalysis (v1.1, NanoTemper Technologies), and Western blot images were further analyzed with Image Lab (v6, Bio-Rad).

For manuscripts utilizing custom algorithms or software that are central to the research but not yet described in published literature, software must be made available to editors and reviewers. We strongly encourage code deposition in a community repository (e.g. GitHub). See the Nature Portfolio guidelines for submitting code & software for further information.

Data

Policy information about availability of data

All manuscripts must include a data availability statement. This statement should provide the following information, where applicable:

- Accession codes, unique identifiers, or web links for publicly available datasets
- A description of any restrictions on data availability
- For clinical datasets or third party data, please ensure that the statement adheres to our policy

Provide your data availability statement here.

Research involving human participants, their data, or biological material

Policy information about studies with human participants or human data. See also policy information about sex, gender (identity/presentation), and sexual orientation and race, ethnicity and racism.

Reporting on sex and gender	*Use the terms sex (biological attribute) and gender (shaped by social and cultural circumstances) carefully in order to avoid confusing both terms. Indicate if findings apply to only one sex or gender; describe whether sex and gender were considered in study design; whether sex and/or gender was determined based on self-reporting or assigned and methods used. Provide in the source data disaggregated sex and gender data, where this information has been collected, and if consent has been obtained for sharing of individual-level data; provide overall numbers in this Reporting Summary. Please state if this information has not been collected. Report sex- and gender-based analyses where performed, justify reasons for lack of sex- and gender-based analysis.*
Reporting on race, ethnicity, or other socially relevant groupings	*Please specify the socially constructed or socially relevant categorization variable(s) used in your manuscript and explain why they were used. Please note that such variables should not be used as proxies for other socially constructed/relevant variables (for example, race or ethnicity should not be used as a proxy for socioeconomic status). Provide clear definitions of the relevant terms used, how they were provided (by the participants/respondents, the researchers, or third parties), and the method(s) used to classify people into the different categories (e.g. self-report, census or administrative data, social media data, etc.) Please provide details about how you controlled for confounding variables in your analyses.*
Population characteristics	*Describe the covariate-relevant population characteristics of the human research participants (e.g. age, genotypic information, past and current diagnosis and treatment categories). If you filled out the behavioural & social sciences study design questions and have nothing to add here, write "See above."*
Recruitment	*Describe how participants were recruited. Outline any potential self-selection bias or other biases that may be present and how these are likely to impact results.*
Ethics oversight	*Identify the organization(s) that approved the study protocol.*

Note that full information on the approval of the study protocol must also be provided in the manuscript.

Field-specific reporting

Please select the one below that is the best fit for your research. If you are not sure, read the appropriate sections before making your selection.

☒ Life sciences ☐ Behavioural & social sciences ☐ Ecological, evolutionary & environmental sciences

For a reference copy of the document with all sections, see nature.com/documents/nr-reporting-summary-flat.pdf

Life sciences study design

All studies must disclose on these points even when the disclosure is negative.

Sample size	Sample sizes were not determined using formal statistical methods; instead, they were selected based on our extensive experience with assay variability and the ability to reliably detect meaningful differences between treatments. Across Western blotting, flow cytometry (including protein uptake, degradation, and surface expression assays), and reporter assays, we have consistently observed low variability, supporting the chosen sample sizes.
Data exclusions	No data were excluded in this study.
Replication	All experiments were successfully reproduced across multiple biological replicates, as detailed in the figure legends.
Randomization	No randomization was required in this study as the primary focus is set on biochemical characterization and in vitro assays.
Blinding	No blinding was required in this study as the primary focus is set on biochemical characterization and in vitro assays.

Reporting for specific materials, systems and methods

We require information from authors about some types of materials, experimental systems and methods used in many studies. Here, indicate whether each material, system or method listed is relevant to your study. If you are not sure if a list item applies to your research, read the appropriate section before selecting a response.

Materials & experimental systems

n/a	Involved in the study
☐	☒ Antibodies
☐	☒ Eukaryotic cell lines
☐	☐ Palaeontology and archaeology
☐	☐ Animals and other organisms
☐	☐ Clinical data
☐	☐ Dual use research of concern
☐	☐ Plants

Methods

n/a	Involved in the study
☐	☐ ChIP-seq
☐	☒ Flow cytometry
☐	☐ MRI-based neuroimaging

Antibodies

Antibodies used

Rabbit anti-ASGR1 (PA580356, Invitrogen, 1:1000)
Mouse anti-GAPDH CL®594 (60004, Proteintech, 1:10000)
Goat anti-human IgG HRP (A18811, Invitrogen, 1:1000)
Mouse anti-His Tag HRP (MA180218, Invitrogen, 1:5000)
Goat anti-rabbit AF™488 (A11008, Invitrogen, 1:1000)
Donkey anti-rabbit CF®680 (20418, Biotium, 1:1000)

Validation

All antibodies were used for applications validated by antibody suppliers per quality assurance provided by each supplier.

Rabbit anti-ASGR1 (PA580356, Invitrogen) is validated for Western Blot, Immunohistochemistry (Paraffin), ELISA and Immunoprecipitation. Manufacturer information and validation details can be found on the product page: https://www.thermofisher.com/antibody/product/ASGR1-Antibody-Polyclonal/PA5-80356

Mouse anti-GAPDH CL®594 (60004, Proteintech) is validated for Western Blot and Flow Cytometry. Specificity was confirmed through multiple experimental applications, and the antibody has been cited in numerous publications. Details are available at https://www.ptglab.com/products/GAPDH-Antibody-CL594-60004.htm.

Goat anti-human IgG HRP (A18811, Invitrogen) is validated for Western Blot, Immunohistochemistry (IHC), and ELISA. It is affinity-purified and cross-adsorbed to reduce cross-reactivity. Manufacturer validation and datasheets are available at https://www.thermofisher.com/antibody/product/Goat-anti-Human-IgG-H-L-Cross-Adsorbed-Secondary-Antibody-Polyclonal/A18811.

Mouse anti-His Tag HRP (MA180218, Invitrogen) recognizes proteins containing the 6x-His motif and is validated for Western Blot and ELISA. It is suitable for detecting His-tagged recombinant proteins. Validation details are provided by the manufacturer at https://www.thermofisher.com/antibody/product/6x-His-Tag-Antibody-clone-AD1-1-10-Monoclonal/MA1-80218.

Goat anti-rabbit AF™488 (A11008, Invitrogen) is validated for Immunocytochemistry (IF) and Flow Cytometry. It is cross-adsorbed to reduce cross-reactivity with human, mouse, and bovine proteins. Full manufacturer validation information is available at https://www.thermofisher.com/antibody/product/Goat-anti-Rabbit-IgG-H-L-Cross-Adsorbed-Secondary-Antibody-Polyclonal/A-11008.

Donkey anti-rabbit CF®680 (20418, Biotium) is validated for ELISA, Flow cytometry, IHC, IF, Western blot. It is highly cross-adsorbed to reduce cross-reactivity with serum proteins from human, mouse, rat, bovine, goat, horse, chicken, hamster, and sheep. Full manufacturer validation information is available at https://biotium.com/product/donkey-anti-rabbit-igg-hl-highly-cross-adsorbed-cf-dye-storm/?attribute_pa_conjugation=cf680.

Eukaryotic cell lines

Policy information about cell lines and Sex and Gender in Research

Cell line source(s)

Expi293F™ cells were obtained from Thermo Fisher, HepG2 cells were obtained from DSMZ (ACC180), and HEK-Blue™ IL-6 Cells from InvivoGen.

Authentication

Cell lines were authenticated by the supplier.

Mycoplasma contamination

Cell lines were verified to be mycoplasma negative by Mycoplasma PCR Detection Kit (G239, Applied Biological Materials Inc. (abm)).

Commonly misidentified lines
(See ICLAC register)

No misidentified lines were used in this study

Palaeontology and Archaeology

Specimen provenance	*Provide provenance information for specimens and describe permits that were obtained for the work (including the name of the issuing authority, the date of issue, and any identifying information). Permits should encompass collection and, where applicable, export.*
Specimen deposition	*Indicate where the specimens have been deposited to permit free access by other researchers.*
Dating methods	*If new dates are provided, describe how they were obtained (e.g. collection, storage, sample pretreatment and measurement), where they were obtained (i.e. lab name), the calibration program and the protocol for quality assurance OR state that no new dates are provided.*

☐ Tick this box to confirm that the raw and calibrated dates are available in the paper or in Supplementary Information.

Ethics oversight	*Identify the organization(s) that approved or provided guidance on the study protocol, OR state that no ethical approval or guidance was required and explain why not.*

Note that full information on the approval of the study protocol must also be provided in the manuscript.

Animals and other research organisms

Policy information about studies involving animals; ARRIVE guidelines recommended for reporting animal research, and Sex and Gender in Research

Laboratory animals	*For laboratory animals, report species, strain and age OR state that the study did not involve laboratory animals.*
Wild animals	*Provide details on animals observed in or captured in the field; report species and age where possible. Describe how animals were caught and transported and what happened to captive animals after the study (if killed, explain why and describe method; if released, say where and when) OR state that the study did not involve wild animals.*
Reporting on sex	*Indicate if findings apply to only one sex; describe whether sex was considered in study design, methods used for assigning sex. Provide data disaggregated for sex where this information has been collected in the source data as appropriate; provide overall numbers in this Reporting Summary. Please state if this information has not been collected. Report sex-based analyses where performed, justify reasons for lack of sex-based analysis.*
Field-collected samples	*For laboratory work with field-collected samples, describe all relevant parameters such as housing, maintenance, temperature, photoperiod and end-of-experiment protocol OR state that the study did not involve samples collected from the field.*
Ethics oversight	*Identify the organization(s) that approved or provided guidance on the study protocol, OR state that no ethical approval or guidance was required and explain why not.*

Note that full information on the approval of the study protocol must also be provided in the manuscript.

Clinical data

Policy information about clinical studies

All manuscripts should comply with the ICMJE guidelines for publication of clinical research and a completed CONSORT checklist must be included with all submissions.

Clinical trial registration	*Provide the trial registration number from ClinicalTrials.gov or an equivalent agency.*
Study protocol	*Note where the full trial protocol can be accessed OR if not available, explain why.*
Data collection	*Describe the settings and locales of data collection, noting the time periods of recruitment and data collection.*
Outcomes	*Describe how you pre-defined primary and secondary outcome measures and how you assessed these measures.*

Dual use research of concern

Policy information about dual use research of concern

Hazards

Could the accidental, deliberate or reckless misuse of agents or technologies generated in the work, or the application of information presented in the manuscript, pose a threat to:

No	Yes	
☒	☐	Public health
☒	☐	National security
☒	☐	Crops and/or livestock
☒	☐	Ecosystems
☒	☐	Any other significant area

Experiments of concern

Does the work involve any of these experiments of concern:

No	Yes	
☒	☐	Demonstrate how to render a vaccine ineffective
☒	☐	Confer resistance to therapeutically useful antibiotics or antiviral agents
☒	☐	Enhance the virulence of a pathogen or render a nonpathogen virulent
☒	☐	Increase transmissibility of a pathogen
☒	☐	Alter the host range of a pathogen
☒	☐	Enable evasion of diagnostic/detection modalities
☒	☐	Enable the weaponization of a biological agent or toxin
☒	☐	Any other potentially harmful combination of experiments and agents

Plants

Seed stocks — *Report on the source of all seed stocks or other plant material used. If applicable, state the seed stock centre and catalogue number. If plant specimens were collected from the field, describe the collection location, date and sampling procedures.*

Novel plant genotypes — *Describe the methods by which all novel plant genotypes were produced. This includes those generated by transgenic approaches, gene editing, chemical/radiation-based mutagenesis and hybridization. For transgenic lines, describe the transformation method, the number of independent lines analyzed and the generation upon which experiments were performed. For gene-edited lines, describe the editor used, the endogenous sequence targeted for editing, the targeting guide RNA sequence (if applicable) and how the editor was applied.*

Authentication — *Describe any authentication procedures for each seed stock used or novel genotype generated. Describe any experiments used to assess the effect of a mutation and, where applicable, how potential secondary effects (e.g. second site T-DNA insertions, mosiacism, off-target gene editing) were examined.*

ChIP-seq

Data deposition

☐ Confirm that both raw and final processed data have been deposited in a public database such as GEO.

☐ Confirm that you have deposited or provided access to graph files (e.g. BED files) for the called peaks.

Data access links
May remain private before publication. — *For "Initial submission" or "Revised version" documents, provide reviewer access links. For your "Final submission" document, provide a link to the deposited data.*

Files in database submission — *Provide a list of all files available in the database submission.*

Genome browser session
(e.g. UCSC) — *Provide a link to an anonymized genome browser session for "Initial submission" and "Revised version" documents only, to enable peer review. Write "no longer applicable" for "Final submission" documents.*

Methodology

Replicates — *Describe the experimental replicates, specifying number, type and replicate agreement.*

Sequencing depth — *Describe the sequencing depth for each experiment, providing the total number of reads, uniquely mapped reads, length of reads and whether they were paired- or single-end.*

Antibodies — *Describe the antibodies used for the ChIP-seq experiments; as applicable, provide supplier name, catalog number, clone name, and lot number.*

Peak calling parameters — *Specify the command line program and parameters used for read mapping and peak calling, including the ChIP, control and index files used.*

Data quality | *Describe the methods used to ensure data quality in full detail, including how many peaks are at FDR 5% and above 5-fold enrichment.*

Software | *Describe the software used to collect and analyze the ChIP-seq data. For custom code that has been deposited into a community repository, provide accession details.*

Flow Cytometry

Plots

Confirm that:

☒ The axis labels state the marker and fluorochrome used (e.g. CD4-FITC).

☒ The axis scales are clearly visible. Include numbers along axes only for bottom left plot of group (a 'group' is an analysis of identical markers).

☒ All plots are contour plots with outliers or pseudocolor plots.

☒ A numerical value for number of cells or percentage (with statistics) is provided.

Methodology

Sample preparation

Surface Staining: HepG2 cells were harvested with Accutase® and transferred to a 96-well V-bottom plate. Cells were washed three times with MACS buffer (PBS containing 2 mM EDTA and 0.5% BSA) and incubated with primary antibody (anti-ASGPR, 1:1000 in MACS buffer) for 30 minutes on ice. Following three washes with MACS buffer, cells were incubated with Alexa Fluor 488-conjugated secondary antibody (1:1000) for 30 minutes on ice in the dark. After three additional washes, cells were stained with Near-IR live/dead dye (1:1000 in 1x PBS) for 5 minutes on ice in the dark. Cells were then washed twice with 150 µL MACS buffer and resuspended in 100 µL MACS buffer for acquisition.

Uptake Assay: HepG2 cells were seeded in 96-well plates, with or without siRNA treatment. After 48 hours, cells were washed once with DPBS (with calcium and magnesium) and incubated in 100 µL serum-free Opti-MEM® for 2 hours at 37�°C. For antibody-based BioDeg constructs, Fc receptor blocking was performed for 15 minutes at 37 °C. BioDeg samples were added at 100 nM and incubated for 2 hours at 37 °C. Cells were washed twice with DPBS containing 2 mM EDTA, detached with Accutase® for 10 minutes at 37 °C, and stained with Near-IR live/dead dye (1:1000 in DPBS) for 5 minutes on ice in the dark. After two washes with MACS buffer, cells were resuspended in 100 µL MACS buffer for flow cytometry analysis.

Instrument | MACSQuant® Analyzer 10

Software | FlowJo™ v10.1

Cell population abundance | N/A

Gating strategy | Initial gating was performed using forward scatter (FSC) and side scatter (SSC) to exclude debris and identify the main cell population. Singlet discrimination was applied via FSC-A vs. FSC-H gating to eliminate doublets and aggregates. Viability gating based on NIR (LIVE/DEAD™Kit, L10119, Invitrogen) staining allowed selection of living cells.

☒ Tick this box to confirm that a figure exemplifying the gating strategy is provided in the Supplementary Information.

Magnetic resonance imaging

Experimental design

Design type | *Indicate task or resting state; event-related or block design.*

Design specifications | *Specify the number of blocks, trials or experimental units per session and/or subject, and specify the length of each trial or block (if trials are blocked) and interval between trials.*

Behavioral performance measures | *State number and/or type of variables recorded (e.g. correct button press, response time) and what statistics were used to establish that the subjects were performing the task as expected (e.g. mean, range, and/or standard deviation across subjects).*

Acquisition

Imaging type(s) | *Specify: functional, structural, diffusion, perfusion.*

Field strength | *Specify in Tesla*

Sequence & imaging parameters | *Specify the pulse sequence type (gradient echo, spin echo, etc.), imaging type (EPI, spiral, etc.), field of view, matrix size, slice thickness, orientation and TE/TR/flip angle.*

Area of acquisition | *State whether a whole brain scan was used OR define the area of acquisition, describing how the region was determined.*

Diffusion MRI ☐ Used ☐ Not used

Preprocessing

Preprocessing software	*Provide detail on software version and revision number and on specific parameters (model/functions, brain extraction, segmentation, smoothing kernel size, etc.).*
Normalization	*If data were normalized/standardized, describe the approach(es): specify linear or non-linear and define image types used for transformation OR indicate that data were not normalized and explain rationale for lack of normalization.*
Normalization template	*Describe the template used for normalization/transformation, specifying subject space or group standardized space (e.g. original Talairach, MNI305, ICBM152) OR indicate that the data were not normalized.*
Noise and artifact removal	*Describe your procedure(s) for artifact and structured noise removal, specifying motion parameters, tissue signals and physiological signals (heart rate, respiration).*
Volume censoring	*Define your software and/or method and criteria for volume censoring, and state the extent of such censoring.*

Statistical modeling & inference

Model type and settings	*Specify type (mass univariate, multivariate, RSA, predictive, etc.) and describe essential details of the model at the first and second levels (e.g. fixed, random or mixed effects; drift or auto-correlation).*
Effect(s) tested	*Define precise effect in terms of the task or stimulus conditions instead of psychological concepts and indicate whether ANOVA or factorial designs were used.*

Specify type of analysis: ☐ Whole brain ☐ ROI-based ☐ Both

Statistic type for inference (See Eklund et al. 2016)	*Specify voxel-wise or cluster-wise and report all relevant parameters for cluster-wise methods.*
Correction	*Describe the type of correction and how it is obtained for multiple comparisons (e.g. FWE, FDR, permutation or Monte Carlo).*

Models & analysis

n/a	Involved in the study
☐	☒ Functional and/or effective connectivity
☒	☐ Graph analysis
☒	☐ Multivariate modeling or predictive analysis

Functional and/or effective connectivity	Immunofluorescence microscopy data processing was carried out using Fiji(ImageJ), with batch analysis applied to ensure consistent settings across all datasets. Quantitative co-localization analysis was conducted using the JACoP plugin, with manual thresholding. Pearson's correlation and Manders' coefficients were calculated for images containing more than 10 cells to ensure statistical robustness.
